# Investigating ICESat-2 ATL08 Terrain Height Estimation Performance and Affecting Factors: The Impact of Land Cover, Slope, and Acquisition Time

**DOI:** 10.3390/s26082485

**Published:** 2026-04-17

**Authors:** Emre Akturk, Arif Oguz Altunel, Samet Dogan

**Affiliations:** 1 Department of Nature Conservation and Biodiversity Management, Faculty of Forestry, Kastamonu University, Kastamonu 37150, Türkiye; 2 Geographic Information Systems and Remote Sensing Research and Application Center, Kastamonu University, Kastamonu 37150, Türkiye; 3 Department of Forest Engineering, Faculty of Forestry, Kastamonu University, Kastamonu 37150, Türkiye; aoaltunel@kastamonu.edu.tr; 4 Architecture and Urban Planning, Mapping and Cadastre Program, İhsangazi Vocational School, Kastamonu University, Kastamonu 37150, Türkiye; sametdogan@kastamonu.edu.tr

**Keywords:** ICESat-2, ATL08, ATLAS, DEM, terrain height, IQR, altimetry, lidar

## Abstract

**Highlights:**

**What are the main findings?**

- The ICESat-2 ATL08 *h_te_best_fit* terrain height metric yielded the lowest validation error (*RMSE* = 3.37 m) with a slight negative *bias* (−0.42 m) relative to terrestrial GNSS reference data in rugged and forested terrain.
- The terrain slope emerged as a prominent factor in the univariate analysis, degrading accuracy due to pulse broadening, followed by dense forest cover, which limits ground photon retrieval.

**What are the implications of the main findings?**

- Implementing stricter quality control, specifically using Interquartile Range (IQR)-based outlier exclusion and higher ground photon thresholds, appeared to significantly improve data reliability in complex environments.
- While ATL08 offers valuable global elevation data, direct engineering applications in mountainous regions require post-processing to mitigate slope-induced systematic underestimation.

**Abstract:**

Spaceborne LiDAR systems, such as ICESat-2, provide critical data for global land cover and topography; however, their performance in rugged, vegetated landscapes requires rigorous local validation. This study evaluates the vertical accuracy of ICESat-2 ATL08 terrain height metrics in the complex Turkish Western Black Sea region, utilizing a reference dataset of high-precision terrestrial GNSS measurements. Following strict IQR-based outlier detection and photon density filtering, 1637 spatially matched segments were analyzed. The *h_te_best_fit* terrain height metric showed the best agreement with the terrestrial GNSS reference data, yielding an *RMSE* of 3.37 m and a mean *bias* of −0.42 m, indicating a slight underestimation of the terrain surface. The univariate analysis revealed a strong positive correlation between terrain slope and vertical error, indicating that slope is the prominent degradation factor contributing to pulse broadening. Additionally, dense forest cover was found to limit ground photon retrieval, leading to increased error margins, whereas nighttime acquisitions offered slightly improved precision. These findings suggest that while ATL08 is a valuable topographic source, slope-dependent corrections are essential for applications in mountainous environments.

## 1. Introduction

Global Digital Elevation Models (DEMs) have advanced rapidly since the release of the Shuttle Radar Topography Mission (SRTM) data in 2000, followed by subsequent products derived from different acquisition technologies and modeling approaches, including TanDEM-X, AW3D30, COPERNICUS, and FABDEM [1,2,3]. As these datasets became widely available, their accuracy and practical applicability were extensively evaluated using a range of validation methods, from topographic maps to field-based measurements. In parallel, the development of laser-based remote sensing technologies, particularly LiDAR, greatly improved the generation of high-precision elevation reference data. Airborne Laser Scanning (ALS), implemented on aircraft, helicopters, and Unmanned Aerial Vehicles (UAVs), enabled the production of dense point clouds and highly accurate DTMs and DSMs. However, despite its high precision, the broader use of LiDAR-derived data has remained limited by sensor costs and the restricted spatial coverage of airborne platforms [4,5].

Spaceborne LiDAR systems, such as the GEDI and ICESat missions, offer a strong solution to the aforementioned challenges by providing extensive spatial coverage, high temporal resolution, and significantly reduced operational costs [6]. In particular, the Advanced Topographic Laser Altimeter System (ATLAS) onboard the ICESat-2 satellite represents a paradigm shift in the acquisition of terrain height and ground elevation components. ICESat-2’s unique photon-counting LiDAR architecture enables excellent vertical resolution for sampling backscattered signals [7]. Leaving behind the traditional single-pixel or full-waveform footprint concept, this technology enables precise surface-to-ground separation via a 3D sampling cloud constructed from consecutive geolocated photon counts. Furthermore, the open-access policy for both raw data and processed secondary products effectively eliminates financial barriers to sensor acquisition and aerial operations, thereby democratizing access to high-precision LiDAR data for the user community [8].

The ‘Level-3A Land and Vegetation Height’ product, named ATL08, is derived from the ATLAS sensor onboard ICESat-2 and provides estimated elevations for both the terrain (ground) surface and the canopy top along the satellite’s ground track. Utilizing the geolocated photon-level observations from the ATL03 product as its raw input, ATL08 aggregates and summarizes ground and canopy surfaces into fixed 100-m segments [9,10]. The reported elevations within these segments are referenced to the WGS84 ellipsoid and the ITRF2014 terrestrial reference frame [7,8,9]. Due to its extensive coverage and provision of different vertical metrics, ATL08 has become a staple dataset across various scientific fields. In forestry and ecology, it facilitates biomass and carbon modeling [11,12], as well as monitoring of forest degradation and recovery through canopy height metrics and vertical profiling [13,14,15,16]. Thus, the accuracy of the ATL08 product directly affects the reliability and validity of these studies and output products.

Reflecting the significance of the ATL08 product, a considerable number of validation studies have been published in the literature. A study conducted by Neuenschwander et al. (2020) [17] validated ATL08 terrain and canopy height estimates with airborne LiDAR data within the boreal forests of southern Finland. Their analysis revealed a mean terrain height error of −0.07 m and a Root Mean Square Error (*RMSE*) of 0.73 m. Furthermore, the study found that data acquired from strong beams during nighttime summer operations yielded the lowest canopy height errors (*RMSE*: 3.18 m). Consequently, the authors explicitly recommended against using weak beams for terrain height estimation. In a similar study, Dandabathula et al. (2020) [18] assessed the accuracy of ATL08 terrain heights in an arid region of India (Rajasthan) using Differential GPS (DGPS) measurements. Their findings indicated that data derived from strong beams had an RMSE exceeding 12 cm in flat and near-flat topography, with a mean bias approaching zero. More recently, Feng et al. (2023) [19] reported an *RMSE* of 2.35 m for terrain and canopy heights over 100-m segments, identifying terrain slope as the predominant factor influencing accuracy. A broader synthesis of the existing literature suggests that the reported accuracies for ATL08 terrain height data exhibit significant variability, ranging from 12 cm to 2.82 m, depending on the validation methodologies employed and the environmental variables considered [17,18,19,20,21,22,23,24,25,26].

Recent ATL08 validation studies have been conducted across a wide range of environmental settings, including boreal forests [17,19], subtropical and continental forests [26], agricultural plains [20], and mountainous regions with varying topographic complexity [20,21,24]. These studies generally evaluated ATL08’s performance based on factors such as terrain slope, land cover characteristics, and vegetation structure, as well as data-acquisition conditions, including night/day observations, seasonality, snow cover, radiation type (strong/weak), and atmospheric filtering [17,20,21,23]. In relatively flat, open areas, the product was generally tested under low-relief conditions and yielded high-accuracy results [17,18,20]. In contrast, in more complex terrains, particular attention was paid to the effects of steep slopes, dense canopy closures, and limited photon penetration to the ground surface on error rates [19,20,21,24,26]. In addition, some studies have compared the accuracy of ATL08 across different vegetation types, including coniferous, broadleaf, mixed forests, and non-forested areas, and investigated how observation timing (summer/winter) and signal conditions affect field inference performance [21,25]. Although this body of literature has substantially improved understanding of ATL08 performance across diverse environmental conditions, validation studies supported by extensive, high-density terrestrial reference data remain limited, particularly in rugged, densely forested landscapes.

Addressing this need, the present study conducts a comprehensive evaluation of ICESat-2 ATL08 terrain height metrics in the challenging terrain of the Turkish Western Black Sea region, using a large reference dataset of more than 100,000 terrestrial measurement points derived from Global Navigation Satellite System (GNSS) observations with centimeter-level vertical accuracy [27]. In addition to the standard validation of the *h_te_best_fit* metric, defined as the best-fit terrain elevation at the midpoint of each 100 m segment [28], this study also rigorously assesses the accuracy of the *h_te_mean* and *h_te_interp* metrics. Furthermore, it systematically investigates the influence of ancillary segment variables, including land cover, terrain slope, acquisition time, and terrain photon count, in order to quantify both the magnitude and direction of their effects on vertical accuracy.

## 2. Materials and Methods

### 2.1. Study Area

**Figure 1:**
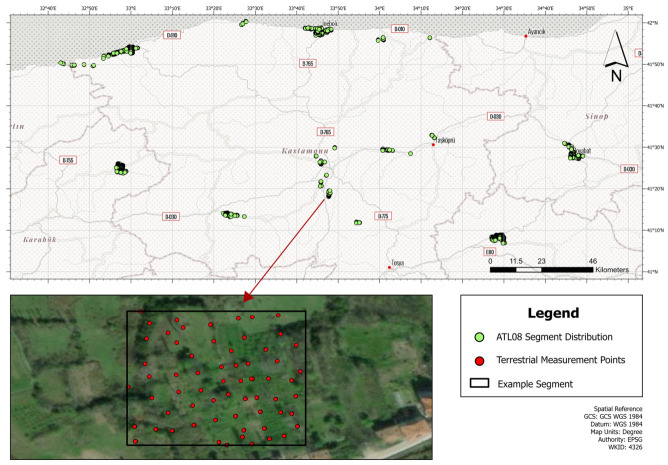
Location of the study area in northwestern Türkiye. The map displays the administrative boundaries of the provinces and the spatial distribution of the ICESat-2 ATL08 segments and collected GNSS measurements in an example segment.

The study area covers the provinces of Kastamonu, Karabük, Bartın, and Sinop, located in the Western Black Sea region, as well as the province of Çorum in the Central Anatolia region of Türkiye (Figure 1). These provinces are characterized not only by their significant contributions to Türkiye’s total forest assets and timber production figures but also by their rugged, complex topography. Kastamonu, which is placed in the center of the designated study area, has more than 60% of its 13,174 km^2^ land area covered with forests [29]. From the Black Sea coast to two high peaks in the south, at 2547 and 2577 m, the provinces’ rolling terrains are considerably varied. Numerous seasonal and permanent streams that originate in or pass through the provinces form basins of varying sizes before emptying into the Black Sea, creating a variety of landform types across the study area. As terrestrial measurements were not conducted continuously across the entire administrative extent of these provinces, this study focuses on a specific sub-province, as illustrated in Figure 1. The spatial distribution of the analyzed segments is particularly concentrated in the coastal sub-province of Kastamonu (Cide, İnebolu, Bozkurt, and Doğanyurt), as well as in the inland sub-province of Araç and the city center of Kastamonu. Additional clusters of segments are located in Eflani, the sub-province of Karabük; Boyabat, the sub-province of Sinop; and Kargı, the sub-province of Çorum, with scattered segments present in various other locations within the studied territory. The irregular spatial distribution of the segments, rather than being confined to a strict administrative boundary, is driven by the spatial intersection between the GNSS-based terrestrial measurement campaign geared specifically for cadastral purposes, conducted between 2020 and 2023, and the corresponding ICESat-2 ATL08 ground tracks. Detailed descriptions regarding data acquisition and processing are provided in Section 2.2 and Section 2.3.

### 2.2. Terrestrial GNSS Data Collection

A critical component of this study is the validation of ATL08 terrain height metrics using high-precision terrestrial measurements. To achieve centimeter-level vertical accuracy, GNSS observations were employed, using multi-frequency data from the GPS, GLONASS, Galileo, and BeiDou constellations, with a primary focus on carrier-phase measurements, which are essential for high-precision geodetic applications [30,31]. Field campaigns were conducted between 2020 and 2023 across the study area. Throughout the survey, measurements were acquired in Real-Time Kinematic (RTK) and Network-RTK (VRS) modes, utilizing constant correction data through TUSAGA-Aktif (CORS-TR), Türkiye’s national continuously operating reference station network. By leveraging reference stations with known coordinates, the network solution delivers real-time models of orbital, clock, and atmospheric errors to the rover receiver. This approach effectively mitigates distance-dependent systematic errors and ensures reliable integer ambiguity resolution for carrier-phase data [32,33].

Field operations were executed using geodetic-grade, (L1/L2) dual-frequency receivers, South surveying system S82 RTK GNSS receiver (Nanjing Hanzhong Mapping Equipment Co., Ltd, Jiangsu, China) equipped with calibrated antennas. Antenna Phase Center Offset (PCO) and Phase Center Variation (PCV) corrections were rigorously applied based on manufacturer calibration files. Following RTK initialization, each point was continuously observed for a minimum of 120 s. Cycle slips were monitored using Melbourne–Wübbena combinations, and observations were re-initialized immediately upon any loss of lock. To maintain robust geometry and signal quality, an elevation mask of 15° was imposed. At the same time, the Position Dilution of Precision (PDOP) was restricted to ≤3, and the signal-to-noise ratio (SNR) was maintained at ≥35 dB-Hz. Multipath effects were practically mitigated by mounting antennas on 2-m poles and selecting sites with open sky visibility, strictly avoiding reflective surfaces such as large metal objects or high building facades [31,32,33]. Connectivity to TUSAGA-Aktif was established via GSM/IP in RTCM format. Error corrections, including ionospheric and tropospheric delays, were handled in real-time via the network’s regional models. Residual tropospheric effects were further minimized using standard delay formulas and appropriate mapping functions consistent with surface elevation and ambient conditions [30,32,34].

Coordinates were derived in an ITRF-96 compliant frame provided by TUSAGA-Aktif. The Turkish National Geoid Model (TR-20) was adopted for converting ellipsoidal heights to orthometric heights. The TG-20 vertical datum is used as a reference for the Turkish National DEM. The Turkish Geoid Model-2020 (TG-20), created by the General Directorate of Mapping (GDM) in Türkiye, is the official geoid model for the nation. TR-20 is a hybrid geoid model that links Türkiye and its near environs between 35°–43° N latitudes and 25°–45° E longitudes. It was created as part of the GDM-coordinated ‘Modernization of the Turkish Height System and Improvement of Gravity Infrastructure (2015–2020)’ project. The SRTM v4.1 digital separation model with a 7.2 arcsecond resolution, a satellite-only global gravity field like GOCO06S and XGM2019e, and over 265,000 terrestrial gravity data and global positioning system (GPS) leveling data were used to create and validate the model [35,36]. The GRS80 ellipsoid is the TG-20 reference ellipsoid [37]. For many uses, the GRS80 and WGS84 ellipsoids are regarded as identical since their differences are negligible [38,39]. GDM provided the TG-20 data, which had a spatial resolution of one arc-minute. Validation GCPs produced over ITRF-96 were translated to ITRF-2014 utilizing the 14-parameter Helmert transformation model as outlined by [40] and [41]. Coordinates were converted to the target epoch utilizing velocity components alongside translation, rotation, and scaling factors [42]. Due to the systematic deviations caused by the error discrepancies between ITRF96 and ITRF2014, especially in the vertical direction, where differences may reach several centimeters [43,44], all GNSS GCPs were transformed to the ITRF2014 system based on the observation epoch. The derived error adjustments were around 1–2 cm vertically and 1 cm horizontally, which were insignificant relative to the current GNSS and ATL08 error uncertainties. Subsequent to the change, all ellipsoidal heights were re-evaluated to conform to the WGS84 reference ellipsoid, guaranteeing complete concordance with the ATL08 data.

Recognizing that geoid selection and grid resolution can introduce centimeter-level systematics into the vertical component, grid specifications were kept uniform throughout the entire dataset. In coastal regions, additional control points were established to constrain boundary-related errors [45,46]. Antenna heights were measured with millimeter precision and correctly entered into the processing software. The entire end-to-end workflow, including transformations from WGS84/ITRF to the national projection, unit processing, and antenna height sign conventions, was standardized to prevent user-induced errors [30,46].

The theoretical error budget accounts for satellite orbit and clock terms, ionospheric and tropospheric delays, receiver-antenna and setup effects, and residuals specific to Network-RTK modeling. Under nominal conditions, network-based RTK sufficiently mitigates baseline-dependent errors, typically achieving a vertical accuracy of approximately 2–3 cm, which aligns with our target precision and published benchmarks [32,45,47]. Spreading measurements over multiple seasons, from 2020 to 2023, helped eliminate seasonal and environmental variability, including fluctuations in ionospheric activity. Sessions that did not meet our observability criteria (PDOP, SNR, and satellite count) or failed to achieve fixed solutions were excluded. Consequently, the resulting set of RTK/Network-RTK heights, quality-filtered and consistently transformed into the orthometric system, provided a robust vertical control dataset against which the accuracy of ATL08 height metrics could be rigorously evaluated [33,34].

### 2.3. ICESat-2 ATL08 Segment Collection and Preprocessing

**Figure 2:**
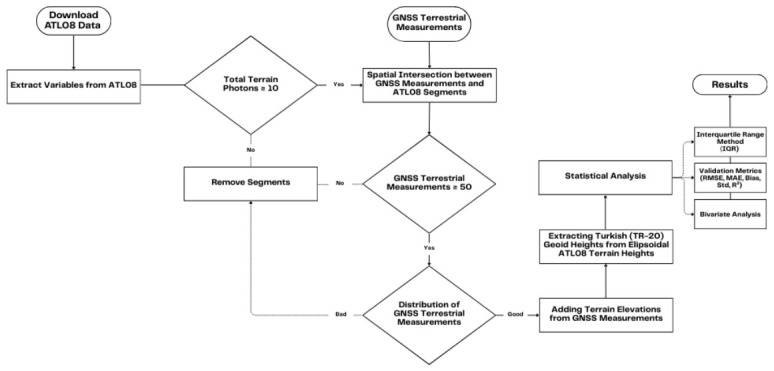
Methodological workflow of the study, illustrating the data acquisition, preprocessing, outlier detection, and statistical validation steps employed for ICESat-2 ATL08 and terrestrial GNSS datasets.

NASA’s Earthdata platform was utilized to acquire ICESat-2 ATL08 data corresponding to the spatial extent of the study area and the temporal span of the terrestrial GNSS campaigns (2020–2023). Specifically, the ATLAS/ICESat-2 L3A Land and Vegetation Height (ATL08) Version 6 (V006) product was employed for this analysis. Data access and retrieval were completed in 2025. To facilitate precise spatial filtering, a shapefile defining the boundaries of the research region was integrated into the Earthdata query system, and all available ATL08 granules falling within the specified timeframe were downloaded to a local repository in HDF5 format. In total, 242 ATL08 granules were retrieved based on these spatial and temporal criteria. To simplify the dataset and exclude variables outside the scope of this study, a Python-based preprocessing script, previously validated in similar research, was used to subset the HDF5 files and retain only the relevant metrics [14,15,48,49]. During this preprocessing phase, only data derived from the high-energy ‘strong’ beams were retained. Weak beams were systematically excluded from the analysis, as previous validation studies have consistently demonstrated the superior performance and higher SNR of strong beams for reliable terrain height estimation [18,19]. Thereafter, the extracted segments from all granules were compiled into a single unified CSV file, thereby preparing the dataset for further filtering and quality control procedures (Figure 2). In elevation studies, it is important to distinguish between Digital Surface Models (DSMs), which represent the uppermost reflective surface including vegetation and built structures, and Digital Terrain Models (DTMs), which aim to represent the bare-earth terrain. The ATL08 variables evaluated in this study are terrain-oriented height metrics and are therefore assessed in the context of terrain/DTM representation rather than DSM-type surface elevation.

Ensuring the quality of the ATL08 segments is a prerequisite for a reliable validation of terrain height metrics. As specified in the ICESat-2 ATL08 Algorithm Theoretical Basis Document (ATBD), the primary criterion for the retrieval of any terrain or canopy parameter is the presence of at least 50 classified photons within a 100-m segment [28]. Consequently, segments failing to meet this minimum photon count threshold were initially excluded. Following the ATBD guidelines, a secondary filter was applied based on the signal-to-noise ratio; segments were discarded if the number of identified ground photons accounted for less than 5% of the total classified photons [28]. Theoretically, this standard criterion implies that a segment with at least 50 photons requires at least 3 ground photons to support terrain metric estimation. However, to ensure a more robust evaluation of terrain quality in this specific study, a stricter threshold was imposed: segments containing fewer than 10 ground photons were systematically excluded from the analysis. This additional limitation primarily affects the lower-density segments (i.e., those with 50–200 total photons), as segments with >200 total photons typically meet the >10 ground photon requirement by default under the 5% rule. Finally, any segments containing invalid or ‘unavailable’ data values (NoData) for the key variables were removed to ensure statistical integrity.

Using the centroidal coordinates of the filtered ATL08 segments, 100 × 100 m vector footprints were generated within the ArcGIS Pro 3.6 version environment. While the effective footprint of the ATLAS beam is significantly narrower across-track (~11 m), the 100 × 100 m buffer is deliberately chosen to evaluate the ATL08 product as a spatial unit comparable to medium-resolution grid cells often used in global topography and biomass modeling. This spatial delineation enabled the spatial intersection of the segments with the underlying terrestrial GNSS measurements. To ensure a rigorous validation framework, a minimum density threshold was implemented, requiring at least 50 random GNSS points to fall within the boundaries of a single segment. Based on this quantitative criterion, a total of 3242 candidate segments were initially identified. However, given that the GNSS dataset was aggregated from various independent field surveys, it was observed that, despite meeting the minimum count of 50, the point distribution in certain segments was spatially heterogeneous or heavily clustered in specific sub-areas. To mitigate potential sampling bias, each of the 3242 segments experienced a manual visual assessment to verify the homogeneity of the GNSS point distribution. Following this detailed monitoring process, 1861 segments were confirmed to exhibit a spatially representative distribution of reference points and were retained for the final comparative analysis (Figure 1).

Following the spatial matching process, the mean elevation value derived from the terrestrial GNSS measurements falling within each segment was appended to the corresponding segment dataset. However, a fundamental prerequisite for any comparative analysis involving altimetry data is the harmonization of vertical datums. All elevation values processed and generated within the ATL08 algorithm are defined relative to the WGS84 ellipsoid [28]. Conversely, in standard engineering and surveying applications in Türkiye, vertical coordinates are predominantly expressed as orthometric heights [50]. In this study, the ellipsoidal heights obtained from GNSS measurements had already been converted to orthometric heights using the local geoid model (TR-20). Therefore, to ensure geodetic consistency and enable direct comparison, the geoid undulation values derived from the TR-20 model were subtracted from the ATL08 terrain height metrics (*h_te_best_fit, h_te_mean*, and *h_te_interp*), thereby transforming them into the same orthometric vertical heights.

### 2.4. Statistical Analysis

Upon completing the data filtering and integration phases, the compiled dataset was subjected to rigorous statistical analysis to compute validation metrics and identify variables that influence the magnitude of error. The analyses in this section were intentionally framed as an exploratory, factor-wise assessment to characterize the observed behavior of ATL08 elevation errors across key environmental and acquisition-related variables, rather than isolating strictly independent effects among potentially interrelated predictors.

As a preliminary step, the Interquartile Range (IQR) method was employed to detect and remove outliers within the dataset. The IQR is widely recognized in descriptive statistics as a robust measure of dispersion, particularly distinguished by its resistance to extreme values compared to methods based on the mean and standard deviation [51]. While the Z-score method is frequently utilized in the literature, the IQR approach was specifically preferred in this study. In the context of satellite altimetry, gross errors can occasionally reach magnitudes of thousands of meters; such extreme outliers would artificially increase the standard deviation, thereby rendering Z-score-based filtering ineffective [52,53]. Conversely, the robust nature of the IQR provides a reliable filtration mechanism by focusing on the middle 50% of the data distribution [51]. The outlier detection thresholds were calculated using Equation (1):

(1) $$IQR=Q_{3}-Q_{1}\;Lower\;Limit=Q_{1}-1.5\times IQR\;Upper\;Limit=Q_{3}+1.5\times IQR$$

where *Q*_1_ represents the first quartile (25th percentile), *Q*_3_ denotes the third quartile (75th percentile), and *IQR* is the calculated range between these two quartiles. Any elevation difference values falling outside the calculated Lower and Upper Limits were classified as outliers and excluded from subsequent accuracy assessments.

To comprehensively quantify the vertical accuracy of the ATL08 terrain height metrics against the reference GNSS data, five fundamental statistical error indicators were employed. First, the Root Mean Square Error (*RMSE*) was calculated as the primary metric for accuracy assessment. *RMSE* is widely regarded as one of the most critical indicators in altimetry validation; it expresses overall accuracy as a single numerical value and, due to the squaring of residuals, assigns higher penalties to larger errors, thus effectively highlighting the presence of outliers [53]. The calculation is given in Equation (2):

(2) $$RMSE=\;\sqrt{\frac{1}{n}\;\sum_{i=1}^{n}{\left(H_{ATL08\left(i\right)}-\;H_{GNSS\left(i\right)}\right)}^{2}}$$

Second, the Mean Absolute Error (*MAE*) was calculated to determine the average magnitude of the vertical discrepancies, disregarding their direction. Unlike *RMSE*, *MAE* measures the average error linearly, treating all deviations equally regardless of their size [54] (Equation (3)):

(3) $$MAE=\;\frac{1}{n}\;\sum_{i=1}^{n}\left|H_{ATL08\left(i\right)}-\;H_{GNSS\left(i\right)}\right|$$

To identify any systematic tendency in the satellite measurements, the Mean Error (*bias*) was evaluated. A positive bias indicates an overall overestimation of terrain height by the ATL08 algorithm, whereas a negative bias suggests underestimation (Equation (4)):

(4) $$Bias=\;\frac{1}{n}\;\sum_{i=1}^{n}\left(H_{ATL08\left(i\right)}-\;H_{GNSS\left(i\right)}\right)$$

Furthermore, the Standard Deviation of the Error (*Std*) was utilized to assess the precision and dispersion of the error distribution around the mean value, independent of systematic offsets [55] (Equation (5)):

(5) $$Std=\;\sqrt{\frac{1}{n-1}\;\sum_{i=1}^{n}{\left(\left(H_{ATL08\left(i\right)}-\;H_{GNSS\left(i\right)}\right)-Bias\right)}^{2}}$$

To further evaluate the precision of the ICESat-2 terrain height metrics and ensure the reliability of the results against potential residual outliers, the Normalized Median Absolute Deviation (*NMAD*) was also calculated. *NMAD* is a robust estimator of the standard deviation, which is less sensitive to outliers in the error distribution [56]. The *NMAD* is defined as follows (Equation (6)):

(6) $$NMAD=\;1.4826\;\times median\;(\left|∆h_{i}-median\;\left(∆h\right)\right|)$$

Finally, the Coefficient of Determination (*R^2^*) was calculated to quantify the strength of the linear correlation and the goodness of fit between the ICESat-2 derived heights and the terrestrial reference measurements [57] (Equation (7)):

(7) $$R^{2}=\;1-\;\frac{\sum_{i=1}^{n}{\left(H_{ATL08\left(i\right)}-\;H_{GNSS\left(i\right)}\right)}^{2}}{\sum_{i=1}^{n}{\left(H_{GNSS\left(i\right)}-\;{\bar{H}}_{GNSS}\right)}^{2}}$$

In these equations, *n* represents the total number of matched segments; $H_{ATL08\left(i\right)}$ indicates the estimated terrain height from the ICESat-2 product for the *i*-th segment; $H_{GNSS\left(i\right)}\;$refers to the corresponding orthometric height obtained from terrestrial GNSS measurements; and ${\bar{H}}_{GNSS}\;$indicates the mean value of the GNSS heights. In addition, $∆h_{i}$ represents the individual vertical errors and $median\;\left(∆h\right)$ is the median of these errors.

Beyond the overall accuracy of the assessment, this study systematically investigated the influence of ancillary parameters, specifically terrain slope (expressed as a percentage by multiplying the ATL08 dimensionless *terrain_slope* ratio by 100), terrain photon count (*n_te_photons*), acquisition time (*night_flag*), and land cover (*segment_landcover*), on the magnitude of vertical errors. To quantify the linear relationship between the continuous variables (terrain slope and terrain photon count) and the elevation differences ∆H, the Pearson Correlation Coefficient (*r*) was calculated, as defined in Equation (8). This metric indicates both the strength and direction of the association between the terrain characteristics and the estimation error [58]. Furthermore, simple linear regression models were fitted to visualize the trend in errors as a function of these continuous variables. For categorical factors, namely land cover classes and acquisition time (day vs. night), the error distributions were examined using boxplots to compare the median discrepancies and interquartile variability across different groups, thereby identifying conditions under which the ATL08 algorithm may exhibit performance degradation:

(8) $$r\;=\;\frac{\sum_{i=1}^{n}\left(x_{i\;}-\;\bar{x}\right)\;\left(y_{i\;}-\;\bar{y}\right)}{\sqrt{\sum_{i=1}^{n}{\left(x_{i\;}-\;\bar{x}\right)}^{2}}\;\sqrt{\sum_{i=1}^{n}{\left(y_{i\;}-\;\bar{y}\right)}^{2}}}$$

where $x_{i\;}$represents the value of the independent variable (e.g., slope or photon count) for the *i*-th segment; $\bar{x}$ is the mean of the independent variable; $y_{i\;}$ is the vertical error (*H_ATL08_* − *H_GNSS_*); and $\bar{y}$ is the mean error.

## 3. Results

### 3.1. Data Quality Control (IQR Method Results)

**Figure 3:**
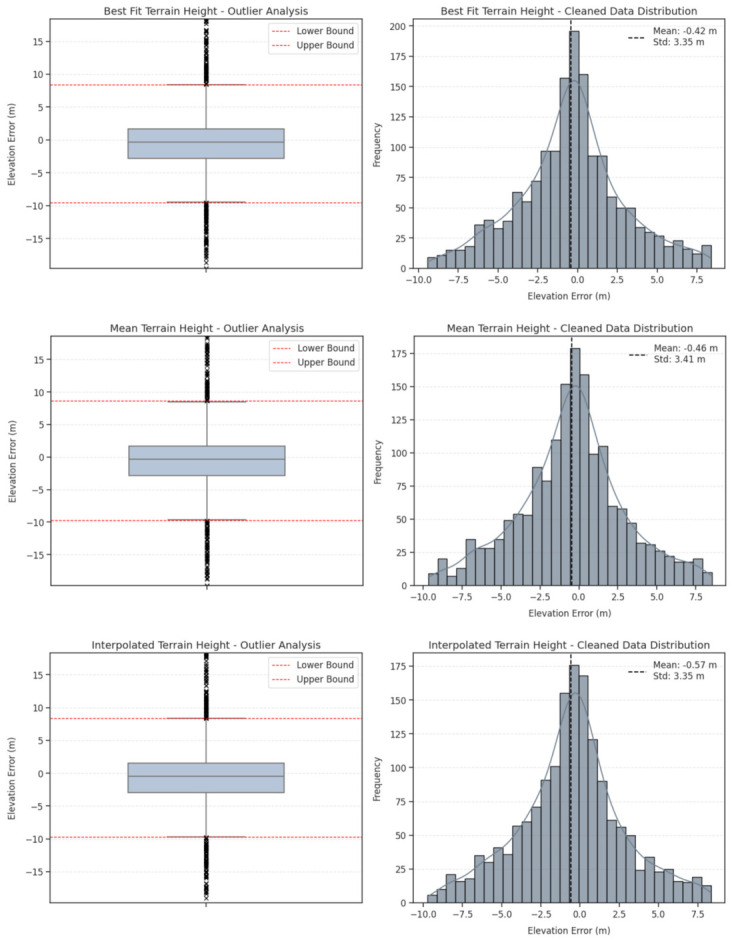
Distribution of elevation differences (∆H= *H_ATL08_* − *H_GNSS_*) for the *h_te_best_fit*, *h_te_mean*, and *h_te_interp*. The left panel illustrates the boxplot of the raw dataset showing extreme outliers, while the right panel displays the histogram of the refined dataset after applying the IQR-based outlier exclusion, overlaid with the mean and standard deviation indicators.

Prior to the fundamental accuracy assessment, the IQR method was applied to the elevation differences (∆H) derived from all three ATL08 terrain height metrics to identify and mitigate the impact of gross errors. The initial dataset consisted of 1861 spatially matched segments for each metric. The analysis revealed that, while the ATL08 algorithm generally performed consistently, extreme deviations occurred due to complex terrain conditions or signal noise. For the primary metric, *h_te_best_fit*, the IQR assessment defined the valid error range between −9.55 m and +8.41 m. Fernandez-Diaz et al. (2022) also stated a similar range while performing a similar study in the North-Central American span [59]. Consequently, 224 segments (approximately 12% of the dataset) were classified as outliers and excluded, resulting in a refined dataset of 1637 segments. Similar outlier detection rates were observed for the other metrics; 216 segments were removed for *h_te_mean*, and 222 segments were removed for *h_te_interp*. The impact of this filtration process on the error distribution is visually presented in Figure 3, and the detailed descriptive statistics for the outlier detection phase are summarized in Table 1. The subsequent accuracy analyses were conducted exclusively on these cleaned datasets to ensure a robust evaluation.

### 3.2. Vertical Accuracy Assessment Results

After outlier removal, the three ICESat-2 ATL08 terrain height metrics were evaluated against terrestrial GNSS reference data to quantify their agreement with point-based ground elevations. A summary of the statistical performance indicators, *RMSE*, *MAE*, *bias*, *Std*, *NMAD*, and *R^2^* is presented in Table 2.

The overall assessment reveals a high degree of agreement between the satellite-derived elevations and the ground validation data, with *R*^2^ values approaching 1.00 across all metrics. Among the evaluated parameters, the *h_te_best_fit* showed the lowest validation, achieving an *RMSE* (3.37 m) and *MAE* (2.53 m) relative to the GNSS reference dataset. Regarding systematic errors, *h_te_best_fit* demonstrated the smallest *bias* of −0.42 m, indicating a slight general underestimation of the terrain surface by the ATL08 algorithm. Comparatively, the *h_te_interp* followed closely, yielding an *RMSE* of 3.40 m and a *bias* of −0.57 m. Conversely, the *h_te_mean* showed slightly higher error margins (*RMSE*: 3.44 m, *bias*: −0.46 m) compared to the other two metrics.

The very high *R*^2^ values obtained in this study mainly reflect the strong correspondence between ATL08-derived and reference elevations over a broad topographic range. They should, therefore, be interpreted as indicators of overall elevation pattern agreement rather than as direct measures of vertical accuracy.

**Figure 4:**
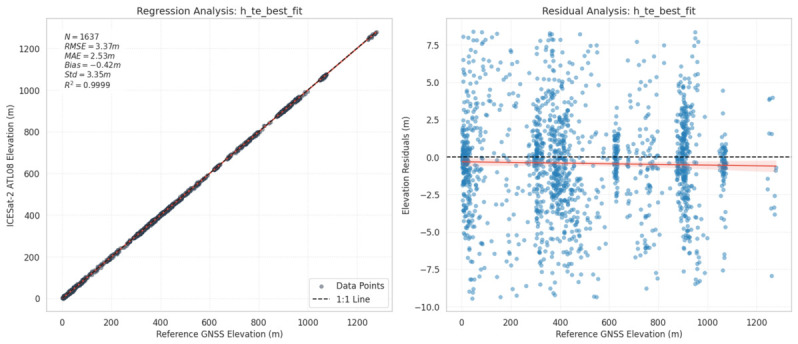
Validation of the ICESat-2 ATL08 h_te_best_fit height metric against GNSS reference data. The left panel displays the linear regression analysis showing the regression fit (red line) and ideal 1:1 correlation (dashed black line), while the right panel illustrates the distribution of vertical errors (∆H) across the elevation range.

The regression and residual analyses for the best-performing metric (*h_te_best_fit*) are visualized in Figure 4. The regression plot (Left Panel) confirms that the estimated elevations are tightly clustered around the 1:1 reference line. The residual plot (right panel) illustrates the distribution of errors around the zero axis, revealing that the majority of discrepancies are concentrated within the ±5 m band and exhibit a random distribution rather than a distinct systematic trend.

### 3.3. Impact of Environmental and Acquisition Factors

**Figure 5:**
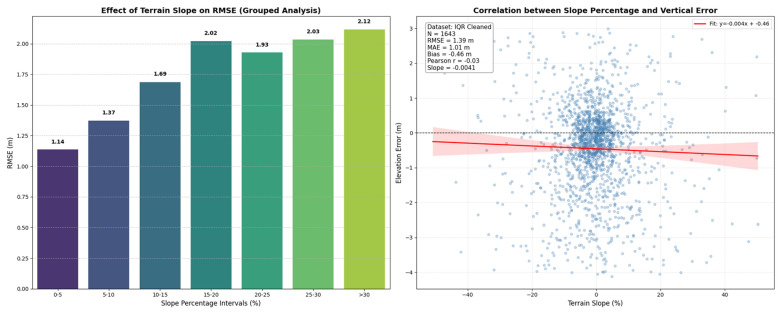
Impact of terrain slope on ICESat-2 ATL08 vertical accuracy. (**Left**) Grouped statistical analysis showing the systematic increase in *RMSE* across slope intervals. (**Right**) Scatter plot of vertical error versus slope percentage, including the fitted linear regression line (red) and its 95% confidence interval (red shaded area); the inset reports key statistical metrics derived from the IQR-excluded dataset.

**Figure 6:**
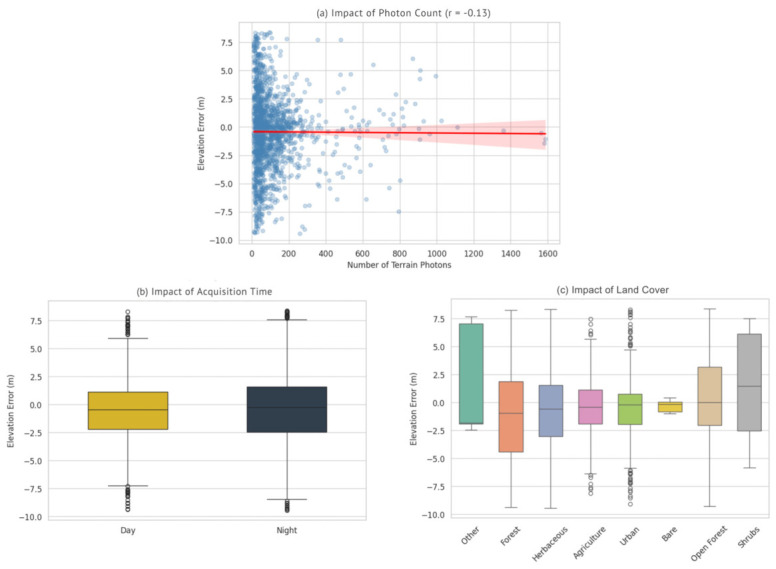
Effects of terrain photon count, acquisition time, and land cover on ICESat-2 ATL08 vertical accuracy. (**a**) Scatter plot showing the relationship between the number of terrain photons and vertical error, with the fitted linear regression line (red) and its 95% confidence interval (red shaded area). (**b**) Boxplot comparing the distribution of vertical errors between daytime and nighttime acquisitions. (**c**) Boxplot illustrating the distribution of vertical errors across different land cover classes.

Beyond the overall accuracy assessment, this study examined the impact of key environmental and acquisition parameters, specifically terrain slope, photon count, acquisition time, and land cover, on the vertical accuracy of the ATL08 product. The relationships between these variables and the elevation errors are visualized in Figure 5 and Figure 6.

As illustrated in Figure 5, while the linear regression analysis of the raw data indicates a weak correlation (*r* = −0.03) due to the high variance inherent in rugged topography, the grouped statistical analysis reveals a distinct and systematic degradation in accuracy associated with an increasing slope. Specifically, this figure demonstrates that the *RMSE* rises progressively from 1.14 m in flat terrain (0–5% slope) to 2.12 m in steep terrain (>30% slope). This substantial increase in *RMSE* confirms that the topographic slope is a prominent factor limiting sensor performance. This phenomenon is attributed to pulse broadening, in which the laser footprint interacts with a larger vertical range of the surface on steep slopes, thereby introducing greater geometric uncertainty and widening the height-error distribution.

Conversely, the relationship between the number of signal photons and elevation error (Figure 6a) appeared weak, suggesting that within the filtered valid range, the photon count alone is not the primary driver of vertical accuracy. This result indicates that the strict filtering criteria applied during the preprocessing phase (removing segments with <10 ground photons) effectively eliminated low-quality data, ensuring that the retained segments possessed sufficient radiometric signal for reliable surface retrieval.

Regarding acquisition time (Figure 6b), the comparison between day and night segments showed slightly better performance during nighttime acquisitions. The boxplots indicate a smaller Interquartile Range for nighttime data, likely due to the absence of solar background noise, which improves the signal-to-noise ratio. However, the median errors for daytime acquisitions remained within acceptable limits, demonstrating that the ATL08 algorithm’s background noise removal and photon classification processes are robust even under high solar ambient noise conditions.

Finally, the impact of land cover (Figure 6c) demonstrated substantial variability in accuracy across different surface types. Segments located in open terrain classes, particularly ‘Agriculture’ and ‘Bare Land’, exhibited the lowest error margins and the most consistent distributions. In contrast, ‘Forest’ classes yielded higher dispersion and larger residuals. This reduced accuracy in forested zones is attributable to the complexity of photon penetration through the canopy and the inherent challenge of accurately resolving the ground surface in dense vegetation compared to open surfaces.

## 4. Discussion and Conclusions

The statistical validation performed in this study revealed that the ICESat-2 ATL08 *h_te_best_fit* terrain height metric showed the lowest validation error relative to the GNSS reference dataset (*RMSE* = 3.37 m; *bias* = −0.42 m), indicating a reasonable level of agreement for terrain height estimation in the rugged and forested landscapes of the Western Black Sea region. To properly contextualize these findings, it is essential to compare them with previous validation efforts conducted across diverse biomes and topographic conditions.

Several studies conducted in low-relief environments have reported sub-meter accuracies, demonstrating the high precision of the ATLAS instrument under optimal conditions. For instance, Dandabathula et al. (2020) validated ATL08 data using DGPS in the arid and flat terrain of Rajasthan, India, achieving an *RMSE* better than 0.12 m with near-zero bias [18]. Similarly, in the boreal forests of Finland, which are characterized by relatively moderate topography, Neuenschwander et al. (2020) and Wang and Liang (2023) reported terrain height *RMSE* values of 0.73 m and 0.72 m, respectively, using airborne lidar as a reference [17,23]. Furthermore, Shang et al. (2022) demonstrated that rigorous filtering strategies can reduce *RMSE* values to approximately 0.3–0.5 m in test sites with favorable conditions [22].

However, validation results in regions with rugged topography and complex vegetation structures, similar to our study area, exhibit higher error margins consistent with our findings. Tian and Shan (2021) provided an essential insight into this topographical variation; while they observed an uncertainty of ~0.2 m in flat terrain, this value surged to ~2.0 m in mountainous areas of the USA [20]. They further noted that steep slopes and large incidence angles tend to cause an underestimation of terrain height, a phenomenon that corroborates the negative bias (−0.42 m) observed in our study. Similarly, Zhu et al. (2022) reported an *RMSE* of 2.82 m (daytime) and 2.23 m (nighttime) in the complex terrains of Spain, noting a severe degradation in accuracy when slopes exceeded 30 degrees [21]. In the North American boreal forests, Feng et al. (2023) evaluated the standard 100 m ATL08 product against LVIS data, obtaining an RMSE of 2.35 m [19]. They identified slope as the dominant factor affecting accuracy.

It should be emphasized that the RMSE of 3.37 m reported in this study does not represent a universal accuracy level for ATL08 under all terrain conditions. Instead, it reflects the agreement between ATL08 terrain height estimates and point-based terrestrial GNSS observations in a predominantly rugged and forested environment, where terrain variability and canopy structure both increase retrieval difficulty. Because ATL08 terrain metrics are summarized over ~100 m segments, part of the reported discrepancy also reflects differences in representation between segment-scale terrain estimation and point-scale ground reference measurements, especially in rough topography [28]. Under flatter, more open-surface conditions, lower error levels would be expected, as indicated by previous validation studies conducted in low-relief, sparsely vegetated landscapes [17,18,31]. In addition, this study specifically evaluates the ATL08 product, a preprocessed, segment-aggregated terrain dataset derived from ATL03 photons. Therefore, while a custom ATL03-based workflow may achieve lower errors in some settings by avoiding the fixed 100 m summarization, such an analysis falls outside the scope of the present study, the purpose of which was to assess the performance of the standard ATL08 terrain product itself.

The *RMSE* of 3.37 m obtained in this study is slightly higher than some of the aforementioned complex-terrain studies. This can be attributed to the extremely rugged characteristics of the Western Black Sea region, combined with dense deciduous and mixed forest cover, which poses a dual challenge for the photon-counting lidar. As highlighted by Dong et al. (2021), high canopy closure combined with steep slopes significantly increases height estimation errors, particularly in tropical-like dense vegetation structures where ground photon retrieval is limited [13]. Additionally, Osama et al. (2024) and Wang et al. (2024) emphasized that seasonal factors, such as snow cover and phenological changes, introduce further variability [24,25]. Such concerns were not detrimental to this study because neither the ground validations nor the investigated ATL08 segments were affected by these mishaps, employing good coordination and meticulous filtering. Considering that our dataset spans multiple seasons and covers a highly heterogeneous landscape, the obtained accuracy indicated that while ATL08 provides valuable topographic data, its direct application in high-precision engineering projects in such rugged terrains requires careful post-processing and bias correction.

Considering the complex topography and vegetation patterns within the study area, terrain slope was identified as the primary factor influencing the accuracy of ATL08. Our analysis results indicated a positive correlation where the magnitude of vertical error increases with the increasing slope angle. This finding is in complete agreement with Feng et al. (2023) and Osama et al. (2024), who identified slope as the most critical parameter affecting accuracy [19,24]. The ‘pulse broadening’ effect, a known phenomenon in laser altimetry, extends the duration of the return signal on steep slopes, thereby introducing uncertainty into height estimation [60]. Furthermore, the negative bias (underestimation of terrain height) observed in our study aligns with the findings reported by Tian and Shan (2021), who noted that high slopes and incidence angles induce a systematic underestimation error in the ATL08 algorithm [20].

In terms of environmental factors, the impact of land cover on accuracy is apparent. While error margins remained minimal in open terrain such as agriculture and bare land, a marked decline in accuracy was observed in forested areas. Dong et al. (2021) emphasized that high canopy closure hinders the detection of ground photons and increases error, particularly in complex forest structures; our findings corroborate this observation [13]. Dense and multi-layered forest structures limit the penetration of laser signals to the ground, which may lead the algorithm to misclassify understory vegetation as the ground surface or cause interpolation errors due to insufficient ground signal return [61].

Regarding the acquisition time and signal strength, it was observed that nighttime measurements exhibited a lower error distribution compared to daytime measurements; however, the difference was not significant enough to hinder operational utility. Zhu et al. (2022) and Neuenschwander et al. (2020) stated that nighttime data yield higher accuracy, which is unaffected by solar background noise [17,21]. The fact that daytime data also maintained acceptable accuracy in our study demonstrates the effectiveness of the ATL08 noise filtering algorithms [48]. Furthermore, the weak relationship observed between photon count and error proves that the requirement for a ‘strong signal/high photon count’ suggested by Dandabathula et al. (2020) and Osama et al. (2024) [18,24] was already satisfied by the strict filtering procedures applied in this study (e.g., the exclusion of segments with <10 ground photons). Therefore, the radiometric quality of the dataset was ensured [18,24].

It should be noted that the spatial matching strategy employed in this study, which utilizes a 100 × 100 m square buffer around the segment centroid, may introduce representativeness errors, particularly in steep terrain. Since the effective across-track footprint of the ICESat-2 beam is narrower than the 100 m buffer width, GNSS points located at the edges of the buffer may reflect elevation values differing from the exact ground track due to slope-induced variations. While the high density of GNSS points helps mitigate this effect by providing a reliable mean surface elevation, future validation efforts could benefit from restricting the validation buffer to a narrower corridor aligned with the ground track, thereby strictly isolating the beam-level performance.

It should also be noted that the factor-based analyses presented here are univariate and provide a description of how ATL08 error processing works. In real-world scenarios, because land cover, land photon count, and acquisition time can vary, the components should be interpreted as independent impact estimates, a point that warrants further investigation in future studies.

While this study provides a further and comprehensive validation of ICESat-2 ATL08 data in a topographically complex region, certain limitations must be acknowledged. A primary constraint is the lack of high temporal resolution of the seasonal metadata for the terrestrial GNSS datasets. Although this study covered a multi-year period, the precise separation of seasonal effects, specifically, the differentiation between snow-covered and snow-free conditions, and leaf-on and leaf-off periods, could not be rigorously quantified due to the intended distribution of the ground control points. Previous research has indicated that snow cover can introduce positive biases in laser altimetry; thus, the inability to isolate this variable represents a notable gap in the current analysis. Additionally, the validation was confined to the *h_te_best_fit* terrain height metric provided in the standard ATL08 product. Future investigations would benefit from processing the raw photon data (ATL03) directly, developing custom noise filtering and photon classification algorithms explicitly tailored for high-slope and dense-canopy environments. Such an approach could mitigate the underestimation errors observed in this study and offer superior accuracy compared to the standard global algorithm.

## Figures and Tables

**Table 1:** Summary of the outlier detection statistics using the IQR method for different ATL08 terrain height metrics.

Terrain Metric	Original Count (*N*)	Lower Limit (m)	Upper Limit (m)	Outliers Removed	Final Count (*N_clean_*)
*h_te_best_fit*	1861	−9.55	8.41	224	1637
*h_te_mean*	1861	−9.76	8.60	216	1645
*h_te_interp*	1861	−9.75	8.34	222	1639

**Table 2:** Comparative statistical accuracy assessment of ICESat-2 ATL08 terrain height metrics against terrestrial GNSS data.

Terrain Metric	*N*	*RMSE* (m)	*MAE* (m)	*Bias* (m)	*Std* (m)	*NMAD* (m)	*R* ^2^
*h_te_best_fit*	1637	3.37	2.53	−0.42	3.35	2.71	0.9999
*h_te_mean*	1645	3.44	2.59	−0.46	3.41	2.80	0.9999
*h_te_interp*	1639	3.40	2.55	−0.57	3.35	2.68	0.9999

## Data Availability

The data presented in this study are available on request from the corresponding author. The specific datasets generated during this research, comprising the spatially matched ICESat-2 ATL08 segments and the corresponding mean orthometric heights derived from terrestrial GNSS measurements, are not publicly available due to privacy restrictions regarding the raw in situ observations.

## Abbreviations

ALS	Airborne Laser Scanning
ASTER	Advanced Spaceborne Thermal Emission and Reflection Radiometer
ATBD	Algorithm Theoretical Basis Document
ATLAS	Advanced Topographic Laser Altimeter System
ATL08	ATLAS Level-3A Land and Vegetation Height
CORS-TR	Continuously Operating Reference Stations-Türkiye
DEM	Digital Elevation Model
DGPS	Differential Global Positioning System
DSM	Digital Surface Model
DTM	Digital Terrain Model
GDEM	Global Digital Elevation Model
GEDI	Global Ecosystem Dynamics Investigation
GNSS	Global Navigation Satellite System
GPS	Global Positioning System
ICESat-2	Ice, Cloud, and Land Elevation Satellite-2
IQR	Interquartile Range
ITRF	International Terrestrial Reference Frame
LiDAR	Light Detection and Ranging
MAE	Mean Absolute Error
PCO	Phase Center Offset
PCV	Phase Center Variation
PDOP	Position Dilution of Precision
RMSE	Root Mean Square Error
RTK	Real-Time Kinematic
SNR	Signal-to-Noise Ratio
SRTM	Shuttle Radar Topography Mission
TR-20	Turkish National Geoid Model
UAV	Unmanned Aerial Vehicle
VRS	Virtual Reference Station
WGS84	World Geodetic System 1984
